# Polymorphisms in the 3′UTR of the *TGF-β1* gene associated with litter size in Ujimqin and Sonid sheep

**DOI:** 10.3389/fvets.2025.1700201

**Published:** 2026-02-11

**Authors:** Shuxin Zhang, Ming Cang, Suhe Alatan, Zhuer Gan, Zhana Naren, He Bu, Guifang Cao, Bin Tong

**Affiliations:** 1 The State Key Laboratory of Reproductive Regulation and Breeding of Grassland Livestock, School of Life Sciences, Inner Mongolia University, Hohhot, China; 2 East Ujimqin Hexig Animal Husbandry Development Company, Xilingol, China; 3 West Ujimqin Banner Altai Agriculture and Animal Husbandry Development Company, Xilingol, China; 4 Sonid Right Banner Sonid Sheep Breeding Technology Company, Xilingol, China; 5 Sonid Left Banner Livestock Germplasm Development Company, Xilingol, China

**Keywords:** *TGF-β1*, litter size, Sonid sheep, Ujimqin sheep, polymorphism

## Abstract

Reproductive characteristics like ovulation frequency and litter number significantly impact the sheep industry. Transforming growth factor beta 1 (*TGF-β1*) functions as a multifunctional regulator of key reproductive processes, plays a pivotal role in mammalian ovarian development. However, research on the role of the *TGF-β1* gene in the reproduction of Ujimqin and Sonid sheep breeds remains scarce. Thus, this study identified 11 new variants of the *TGF-β1* gene in these breeds by direct sequencing. In these 11 variants, the Sonid-linkage disequilibrium (LD)1 and Ujimqin-LD1 (comprising the g.50044287 G > A and g.50044526 G > C polymorphisms) in the 3′ untranslated region of the *TGF-β1* gene were significantly associated with litter size in Sonid and Ujimqin ewes (*p* < 0.01, *p* < 0.05, respectively), and the single g.50044837 C > T polymorphism in the 3′UTR was also associated with litter size in Sonid sheep (*p* < 0.01) through association analyses. These findings may provide potential genetic markers for improving prolificacy in sheep.

## 1 Introduction

Lamb is one of the most widely consumed meats in the world, and its proportion in the Chinese market is also increasing year by year. The rich nutrition and tender taste of lambs are favored by the majority of consumers (1). Meanwhile, the Sonid and Ujimqin sheep, both native to Inner Mongolia, play a crucial role in maintaining the health of northern China’s grasslands and are considered important native genetic resources for local sheep breeds (2, 3). As integral components of China’s indigenous sheep genetic diversity, Sonid and Ujimqin sheep exhibit superior production attributes (including high mutton yield, and premium meat quality) and exceptional adaptation to nomadic herding systems (4). Furthermore, they possess exceptional stress resistance characterized by outstanding drought tolerance and cold hardiness, which constitute decisive adaptive advantages for sustainable livestock production in arid and temperate grassland ecosystems (5). Litter size is a vital economic characteristic in sheep breeding and production. However, Sonid and Ujimqin sheep exhibit low reproduction, with an average lambing number per ewe ranging from only 1.03 to 1.13 lambs (6). Concurrently, reproductive traits is a lowly heritable trait, rendering traditional phenotypic selection strategies ineffective due to limited genetic progress and suboptimal breeding outcomes (7). Therefore, it is imperative to enhance ewe prolificacy by identifying candidate functional genes and mutations associated with litter size in Sonid and Ujimqin sheep breeds, and subsequently utilizing these polymorphisms for marker-assisted selection (MAS) breeding.

With advances in molecular biology, researchers worldwide have conducted extensive studies on genes associated with sheep reproduction and their correlation with litter size, such as bone morphogenetic protein receptor IB (*BMPRIB*), bone morphogenetic protein 15 (*BMP15*), and growth differentiation factor 9 (*GDF9*) (8). These genes all belong to the TGF-*β* superfamily, yet *TGF-β1* has been relatively less studied. The *TGF-β1* (transforming growth factor beta 1) gene, a part of the TGF-β superfamily, is a highly conserved secreted peptide growth factor (9). This superfamily comprises more than 30 structurally similar but functionally diverse members, playing critical roles in various physiological processes such as cell growth and differentiation, immune regulation, and embryonic development (10). *TGF-β1* binds to serine/threonine kinase receptors TβRI (type I TGF-β receptor) and TβRII (type II TGF-β receptor) on the cell membrane surface, initiating the formation of a heteromeric complex that triggers phosphorylation of Smad2/3 (11). Phosphorylated Smad2/3 combine with Smad4 to enter the nucleus and directly control target gene transcription (12). *TGF-β1* simultaneously activates multiple pathways including RAS–ERK and PI3K-AKT, thereby forming a complex signaling network that regulates extracellular matrix remodeling and epithelial-mesenchymal transition (13). These non-classical signaling pathways interact with the Smad signaling pathway to co-regulate essential biological processes such as cell proliferation (14), differentiation, migration (15), and apoptosis (16). Studies have shown that *TGF-β1* knockout causes embryonic lethality (yolk sac vascularization defects), while its overexpression in rats significantly enhances ovulation rates in mice (17, 18). Notably, in Small-tailed Han sheep, TGF-β1 orchestrates ovarian granulosa cell proliferation and apoptosis through both the canonical Smad4-dependent pathway and non-canonical pathways (notably p38 MAPK) during follicular development, while concurrently modulating steroid hormone secretion (19, 20). TGF-β1 enhances follicular development in Hu sheep by promoting granulosa cell proliferation, exhibiting significantly higher expression in ovarian tissue of multiparous ewes versus uniparous ewes, thereby improving litter size and overall reproductive performance (21). In Large White pigs, the haplotype TATGG formed by four polymorphic sites in the intronic region of the *TGF-β1* gene is significantly associated with litter size (22). In goats, two key mutation sites (an A > G mutation at 148 bp in exon 2 and a G > C mutation at 790 bp in intron 2) of the *TGF-β1* gene are significantly correlated with kidding rate (23, 24). These findings collectively establish TGF-β1 as a multifunctional regulator of key reproductive processes in animals. Nevertheless, research on the *TGF-β1* gene in sheep, especially in Sonid and Ujimqin sheep breeds remains scarce.

In addition, a growing body of studies consistently reports that the 3′UTR of mRNA can bind to miRNAs, thereby suppressing gene translation or directly promoting mRNA degradation to regulate gene expression (25–27). miR-134-3p controls the proliferation and apoptosis of sheep granulosa cells (GCs) by modulating the TGF-*β*/PI3K/AKT pathway (28). In Awassi sheep, a polymorphism (319 C > T) in the 3′UTR of the *PITX2* gene is associated with litter size (29). In Small-tail Han sheep, a polymorphism (rs161611767 T > C) in the 3′UTR of the *ETS1* gene is associated with litter size. miR-216a-3p functions as a regulatory element by binding to the T allele of rs161611767, thereby regulating *ETS1* expression, influencing granulosa cell development, and potentially indirectly affecting lambing performance (30). These findings collectively suggest that mutations in the 3′UTR of sheep genes may also influence reproductive performance.

Therefore, this study selected the *TGF-β1* gene as a candidate functional gene. Association analysis was conducted between novel mutations identified in this study and litter size in Sonid and Ujimqin sheep populations. This study seeks to provide marker-assisted selection related to litter size for Sonid and Ujimqin sheep populations. Additionally, it also provides new insights into the impact of the *TGF-β1* gene on reproductive traits in sheep.

## 2 Materials and methods

All animal welfare and experimental protocols adhered to the standards set by the China Ministry of Science and Technology (2004). The research received the green light from the Institutional Animal Care and Use Ethics Committee at Inner Mongolia University (approval No. 93) on May 15, 2015, under the animal experiment permit IMU-2015-03.

### 2.1 Sample and data

Details of the 148 Ujimqin ewes utilized in this study were previously described (31). The 231 Sonid sheep were sourced in 2023 from Sonid Right Banner (Sonid Right Banner Sonid Sheep Breeding Technology Company) and Sonid Left Banner (Sonid Left Banner Livestock Germplasm Development Company) in Inner Mongolia, China. All Sonid sheep in this region are purebred, without genetic introgression from exotic breeds. Based on the lambing records of the aforementioned company, 187 ewes that produced single lambs and 44 ewes that consistently produced twins (over four consecutive lambing seasons) were randomly selected. These selected animals formed the foundation stock for establishing the Sonid sheep pedigree. We confirm that the 231 Sonid and 148 Ujimqin ewes included in this study were unrelated individuals, carefully selected to avoid any kinship. All experimental ewes were maintained under standardized conditions with ad libitum access to feed, water, and natural light. Considering the well-known influence of the *FecB* mutation on sheep litter size, we initially tested the Sonid ewes for this genetic variation by PCR-RFLP (6) and found that none of the animals in our study carried this mutation. Blood samples, each about 10 mL, were drawn from the jugular vein into EDTA-K2 tubes and kept at −20 °C until further testing.

### 2.2 Re-sequencing and variants detection in TGF-β1

With the objective of identifying *TGF-β1* gene polymorphisms, a total of 10 Sonid sheep (including five ewes with single lambs and five with twins) and 10 Ujimqin sheep (five with a single lamb and five with twin lambs) were subjected to direct sequencing. To design primers specific to the *TGF-β1* gene, Primer 5.0 software from Premier Biosoft International (Palo Alto, CA, USA) was used. By referencing the ovine *TGF-β1* DNA sequence (ARS-UI_Ramb_v3.0; the National Center for Biotechnology Information (NCBI) accession: NC_056067.1), the NCBI BLAST tool was employed to amplify all exons, promoters, 5′ flanking region, and 3′ untranslated region (3′UTR) regions of the gene. The Primer 5.0 software package, developed by Premier Biosoft International (Palo Alto, CA, USA), was instrumental in the design of species-specific PCR primers. These primers were engineered to target the *TGF-β1* gene. Drawing upon the ovine *TGF-β1* DNA sequence (ARS-UI_Ramb_v3.0; NCBI reference sequence: NC_056067.1), we leveraged the National Center for Biotechnology Information BLAST tool to ensure comprehensive amplification, encompassing all exons, promoters, the 5′ flanking region, and the 3′UTR of the gene. The established PCR protocol involved a 5 min pre-denaturation step at 95 °C, followed by 35 cycles. Each cycle consisted of a 10 s denaturation at 98 °C, a 5 s annealing period at 58 °C, and a 5 s extension at 68 °C. A final extension was carried out at 72 °C for 10 min. The precise annealing temperatures for each amplified fragment are detailed in Table 1. Subsequently, the PCR products underwent analysis via 3.0% agarose gel electrophoresis to ascertain both the quality and quantity of the DNA for sequencing. The sequencing itself was conducted by the Beijing Genomics Institute (BGI) in Beijing, China.

**Table 1:** PCR primers used for sequencing of *TGF-β1.*

Name	Target region	Primer sequence (5′ → 3′)	Annealing temperature (°C)	Product length (bp)
Promoter-1	Promoter	F: TTGAGCCGAGGAAGAACC	58	891 (−2,246 ~ −1,355 promoter)
		R: TCACCCAGAGTGGAGAAGG		
Promoter-2	Promoter	F: ACGTGGAAGGGCTCAATAA	56	941 (−1,528 ~ −607 promoter)
		R: CAGCGGAAAAGTCTCAAAAC		
5’UTR	5′ flanking region	F: CGAGCTGGTTGGGAGAAGA	60	532 (35 bp promoter + 497 bp 5’flanking region)
		R: CGAGGAAAAGGTAGGAGGGT		
TGF-β1-1	Exon 1	F: ACCCTCCTACCTTTTCCTCG	58	710 (122 bp 5’flanking region + 355 bp exon 1 + 233 bp intron 1)
		R: AAGCGGTCCACTTCACTCAC		
TGF-β1-2	Exon 2	F: GCACGAAGCCCAAAGATC	61	516 (2,411 bp intron 1 + 161 bp exon 2 + 114 bp intron 2)
		R: CTCTGTGCTCCCTCATCCTT		
TGF-β1-3	Exon 3	F: AGGGTGGAGGCTGAAATG	64	609 (234 bp intron 2 + 118 bp exon 3 + 257 bp intron 3)
		R: ACCCAAGCAATGGAGCA		
TGF-β1-4/5	Exon 4/5	F: GCCAAGAACTGGAGCAAGA	58	757 (168 bp intron 3 + 78 bp exon 4 + 153 bp intron 4 + 148 bp exon 5 + 210 bp intron 5)
		R: TCGGCGTTCCACATTCTA		
TGF-β1-6	Exon 6	F: CAAGTTGGATACCGTGCTG	54	412 (107 bp intron 5 + 154 bp exon 6 + 151 bp intron 6)
		R: TCCCCTCCAACCTTCCTC		
TGF-β1-7	Exon 7	F: AAGATGCCAGCAAGGACA	52	424 (220 bp intron 6 + 180 bp exon 7 + 24 bp intron 7)
		R: GGGTCGCAACCGGAGT		
3′UTR-1	3′UTR	F: TGCGACCCACCAGCAGTTAC	52	722 (3′UTR)
		R: TCCCAGGTCCCAGGCACAT		
3′UTR-2	3′UTR	F: GTGCGGTGTCTTTCGTTT	58	536 (3′UTR)
		R: CCACAGAGGCTGAGAACA		
3′UTR-3	3′UTR	F: TGGTATTCTGAGGATGTTTGT	58	881 (3′UTR)
		R: TCTGCTTCCCCATACCTT		

CDS, coding region; F, forward primer sequence; R, reverse primer sequence.

### 2.3 SNP genotyping using iPLEX MassARRAY

Genotyping of 11 new variants was conducted in 231 Sonid sheep and 148 Ujimqin sheep with the MassARRAY® SNP Genotyping System (BioScience, San Diego, CA, USA). Primer pairs specific to the *TGF-β1* gene sequence were designed, and 11 novel variants were genotyped in the 231 Sonid sheep and 148 Ujimqin populations using the MassARRAY SNP genotyping system (Agena Bioscience, San Diego, CA, USA). The PCR and extension primers for *TGF-β1* were designed using Assay Design Suite software^1^ with default parameters, incorporating sequences containing each target mutation along with approximately 100 bp upstream and downstream flanking regions. Allele genotypes were determined using the Sequenom MassARRAY iPLEX Platform, with data analyzed via MassARRAY Typer 4.0 Analyzer software (BioScience, San Diego, CA, USA).

### 2.4 Bioinformatics analysis

The basic characteristics of the predicted TGF-β1 protein were analyzed using ProtParam.^2^ We employed the default threshold of 0.5 for all networks (Serine, Threonine, and Tyrosine). Sites with a score above this threshold were considered potential phosphorylation sites. DeepTMHMM^3^ predicts alpha and beta transmembrane proteins using deep neural networks. Potential phosphorylation sites were predicted using NetPhos 3.1^4^ with the default threshold of 0.5. Serine (Ser), threonine (Thr), and tyrosine (Tyr) residues with scores above this threshold were reported as potential phosphorylation sites. The hydrophobicity (using the Kyte & Doolittle amino acid scale) and flexibility profiles (using the Karplus-Schulz flexibility scale) of the protein were assessed via ProtScale.^5^ N-glycosylation sites were predicted using NetOGlyc-4.0^6^ with its default settings, considering sites with a combined score > 0.5 as potential glycosylation sites. The organization of conserved domains was annotated using the SMART database (version 9.0; http://smart.embl-heidelberg.de/) with default parameters, which identifies functional regions based on sequence similarity. Furthermore, the secondary structure of the mRNA transcript was predicted using the RNAfold web server^7^ under default parameters (temperature = 37 °C, no constraints), providing insights into the minimum free energy (MFE) structure and base-pairing probabilities. Protein secondary structure was predicted using SOPMA^8^ with default parameters (window width: 17; similarity threshold: 8). Homology analysis and phylogenetic tree construction were performed using NCBI BLAST, the UniProt alignment tool,^9^ and MEGA-X software employing the Neighbor-Joining method, Poisson model for evolutionary distance, and 1,000 bootstrap replicates. Additionally, potential microRNA binding sites within the TGF-β1 3′UTR were identified using miRanda (score threshold > 140, free energy threshold < −10 kcal/mol; https://www.Bioinformatics.com.cn/local_miranda_miRNA_target_prediction_120), RNAhybrid (binding site MFE threshold < −20 kcal/mol; http://bibiserv.techfak.uni-bielefeld.de/rnahybrid), and miRBase,^10^ considering both sequence complementarity and thermodynamic stability to ensure accurate prediction.

### 2.5 Statistical analysis

For the populations of 231 Sonid and 148 Ujimqin sheep, analyses were conducted to determine genotype and allele frequencies, assess conformity to Hardy–Weinberg equilibrium, and calculate various population genetics parameters such as observed heterozygosity (H_o_), expected heterozygosity (H_e_), the effective number of alleles (n_e_), and polymorphism information content (PIC). Linkage disequilibrium (LD) between genetic loci was examined using HAPLOVIEW version 4.2, with measurements of D*′* and *r^2^* provided to gauge the extent of non-random association (32). Chi-square tests were applied to analyze allele frequency distributions within each mutation locus (33–35). To examine how different genotypes influence the number of lambing in Sonid and Ujimqin sheep, the statistical linear model was defined as follows: Y_ij_ = *μ* + F_i_ + Gj + e_ij_, where Y_ij_ is the phenotypic value of litter size, μ is the population’s overall mean, F_i_ is the fixed effect of farm, G_j_ is the fixed effect of genotype, and e_ij_ is the random error. Because the sample sizes were limited, any groups with fewer than ten animals were omitted from the statistical analysis (Supplementary Table S2).

## 3 Results

### 3.1 Identification variation of *TGF-β1* gene in Sonid and Ujimqin sheep breeds

**Figure 1:**
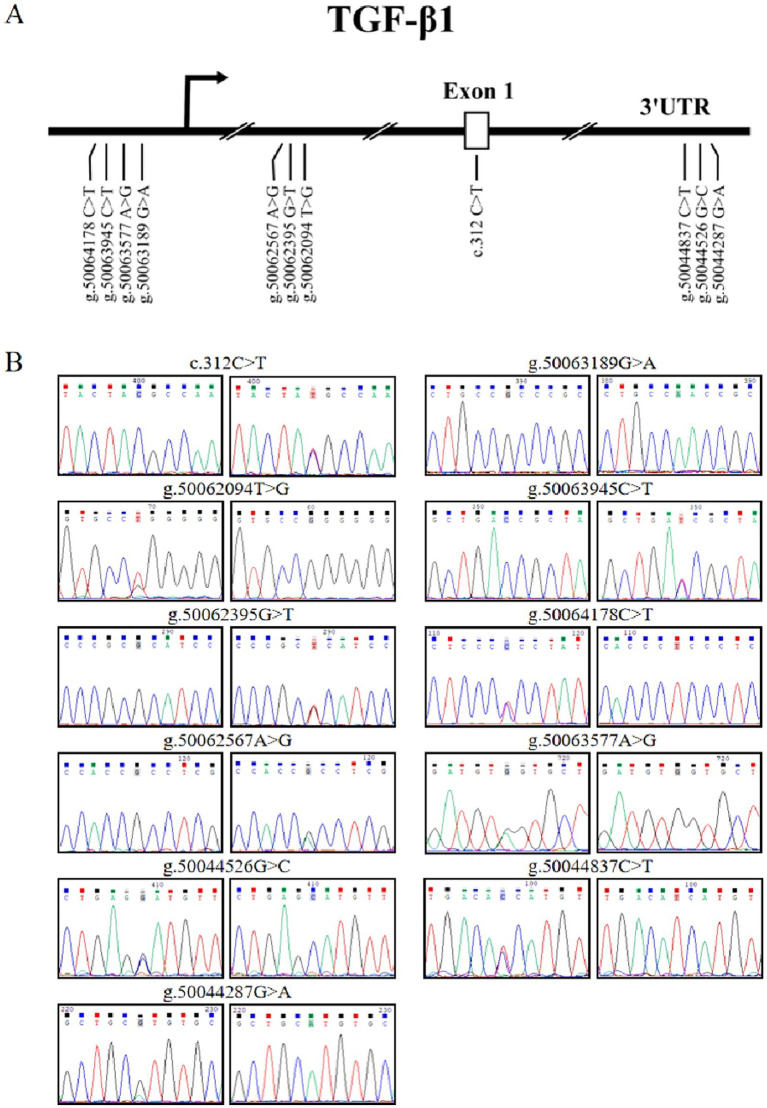
*TGF-β1* variants identified in sheep. **(A)** Physical positions of the 11 variants. **(B)** Nucleotide substitutions. Variants located on chromosome 14 (ARS-UI_Ramb_v3.0; NC_056067.1).

**Figure 2:**
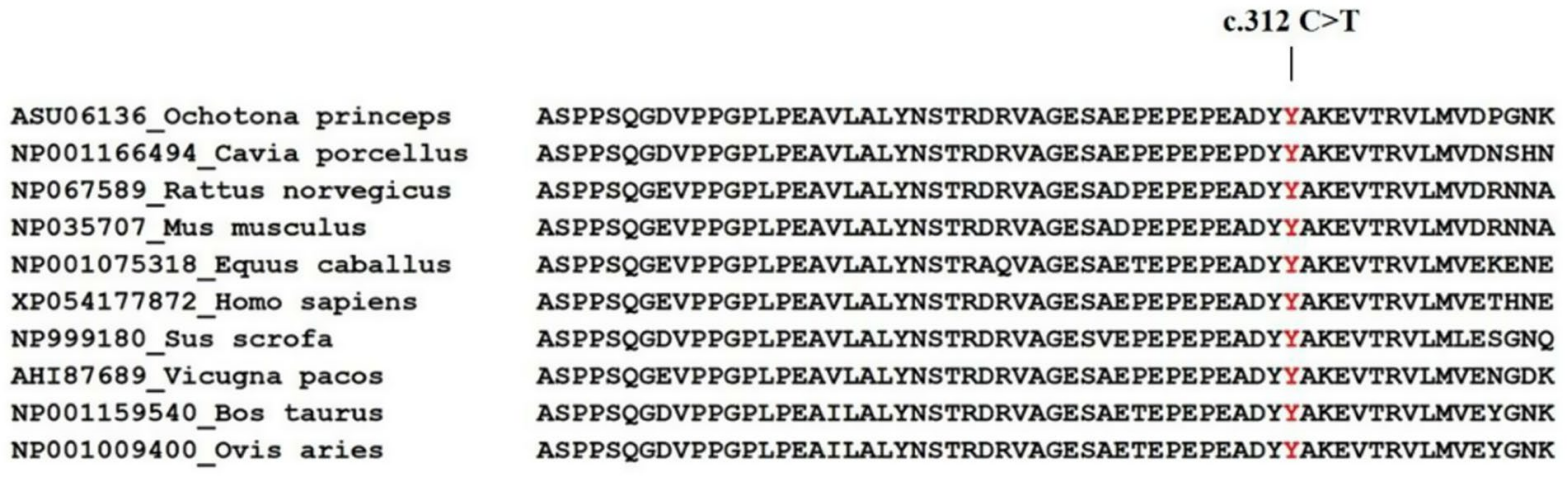
Multi-species amino acid sequence alignment of the TGF-β1 region corresponding to synonymous mutation sites.

Sequence analysis identified two novel and nine known genetic variants in the *TGF-β1* gene across Sonid and Ujimqin sheep breeds. These comprised one exonic synonymous variant (c.312 C>T, ss17090122664), three 5’ flanking region, regulatory variants (g.50062094 T>G (ss17090122663), g.50062395 G>T (rs3498046280), g.50062567 A>G (rs5522182108)), four promoter regulatory variants (g.50063189 G>A (rs594638479), g.50063577 A>G (rs421419500), g.50063945 C>T (rs600009062), g.50064178 C>T (rs408158166)), and three 3’UTR polymorphisms (g.50044287 G>A (rs423387541), g.50044526 G>C (rs411268612), g.50044837 C>T (rs425529296)). The C.312 C>T in exon 1 was synonymously replaced (Figure 1), with the corresponding amino acid (tyrosine) exhibiting high evolutionary conservation among mammals (Figure 2).

### 3.2 Genetic diversity analysis of the *TGF-β1* gene

Genetic variation indices (Ho, He, Ne, PIC) and allele/genotype frequencies for each variant in Sonid and Ujimqin sheep are listed in Table 2. Most variants exhibited moderate polymorphism (0.25 < PIC < 0.5) in both breeds. Except for the mutations c.312 C > T, g.50062094 T > G, g.50062395 G > T, g.50062567 A > G, g.50063577 A > G, g.50063945 C > T, and g.50064178 C > T, which exhibited low polymorphism levels (PIC < 0.25) in both breeds, all other variants demonstrated moderate polymorphism (0.25 < PIC < 0.5) in Sonid and Ujimqin sheep populations.

**Table 2:** Population genetic information of *TGF-β1* SNPs.

SNP locus /Breeds	Genotypic frequencies			Allelic frequency		Ho	He	Ne	PIC	*χ*^2^ (HWE)
c.312 C > T	TT	CT	CC	T	C					
Ujimqin	0.993	0.007	0	0.997	0.003	0.993	0.007	1.007	0.006	0.002
g.50062094 T > G	TT	TG	GG	T	G					
Sonid	0.004	0.056	0.939	0.032	0.968	0.937	0.063	1.067	0.06	2.511
Ujimqin	0	0.061	0.939	0.03	0.97	0.941	0.059	1.063	0.057	0.146
g.50062395 G > T	GG	GT	TT	G	T					
Sonid	0.991	0.009	0	0.996	0.004	0.991	0.009	1.009	0.008	0.004
g.50062567 A > G	AA	AG	GG	A	G					
Sonid	0.026	0.221	0.753	0.136	0.864	0.764	0.236	1.308	0.207	0.907
Ujimqin	0.014	0.25	0.736	0.139	0.861	0.761	0.239	1.313	0.211	0.334
g.50063189 G > A	GG	GA	AA	G	A					
Sonid	0.187	0.443	0.37	0.409	0.591	0.517	0.483	1.935	0.367	1.563
Ujimqin	0.095	0.534	0.372	0.361	0.639	0.538	0.462	1.857	0.355	3.616
g.50063577 A > G	AA	AG	GG	A	G					
Sonid	0.004	0.152	0.844	0.08	0.92	0.853	0.147	1.173	0.136	0.185
Ujimqin	0	0.108	0.892	0.054	0.946	0.898	0.102	1.114	0.097	0.483
g.50063945 C > T	CC	CT	TT	C	T					
Sonid	0.892	0.108	0	0.946	0.054	0.898	0.102	1.114	0.097	0.756
Ujimqin	0.899	0.101	0	0.949	0.051	0.904	0.096	1.106	0.092	0.422
g.50064178 C > T	CC	CT	TT	C	T					
Sonid	0.026	0.197	0.776	0.125	0.875	0.781	0.219	1.28	0.195	2.178
Ujimqin	0.014	0.23	0.757	0.128	0.872	0.776	0.224	1.288	0.198	0.104
g.50044287 G > A	GG	GA	AA	G	A					
Sonid	0.095	0.437	0.468	0.314	0.686	0.569	0.431	1.757	0.338	0.053
Ujimqin	0.095	0.426	0.48	0.307	0.693	0.574	0.426	1.742	0.335	0
g.50044526 G > C	GG	GC	CC	G	C					
Sonid	0.095	0.437	0.468	0.314	0.686	0.569	0.431	1.757	0.338	0.053
Ujimqin	0.095	0.426	0.48	0.307	0.693	0.574	0.426	1.742	0.335	0
g.50044837 C > T	CC	CT	TT	C	T					
Sonid	0.139	0.498	0.364	0.387	0.613	0.525	0.475	1.904	0.362	0.551
Ujimqin	0.142	0.473	0.385	0.378	0.622	0.53	0.47	1.888	0.36	0.004

H_o_: observed heterozygosity; H_e_: expected heterozygosity; n_e_: effective allele numbers; PIC: polymorphism information content; HWE: Hardy–Weinberg equilibrium; The classification was conducted according to the PIC value (PIC value < 0.25, low polymorphism; 0.25 < PIC value < 0.5, moderate polymorphism; and PIC value > 0.5, high polymorphism); There were Hardy–Weinberg deviation from the obtained genotype frequency.

### 3.3 Linkage disequilibrium analysis of novel variants in *TGF-β1*

**Figure 3:**
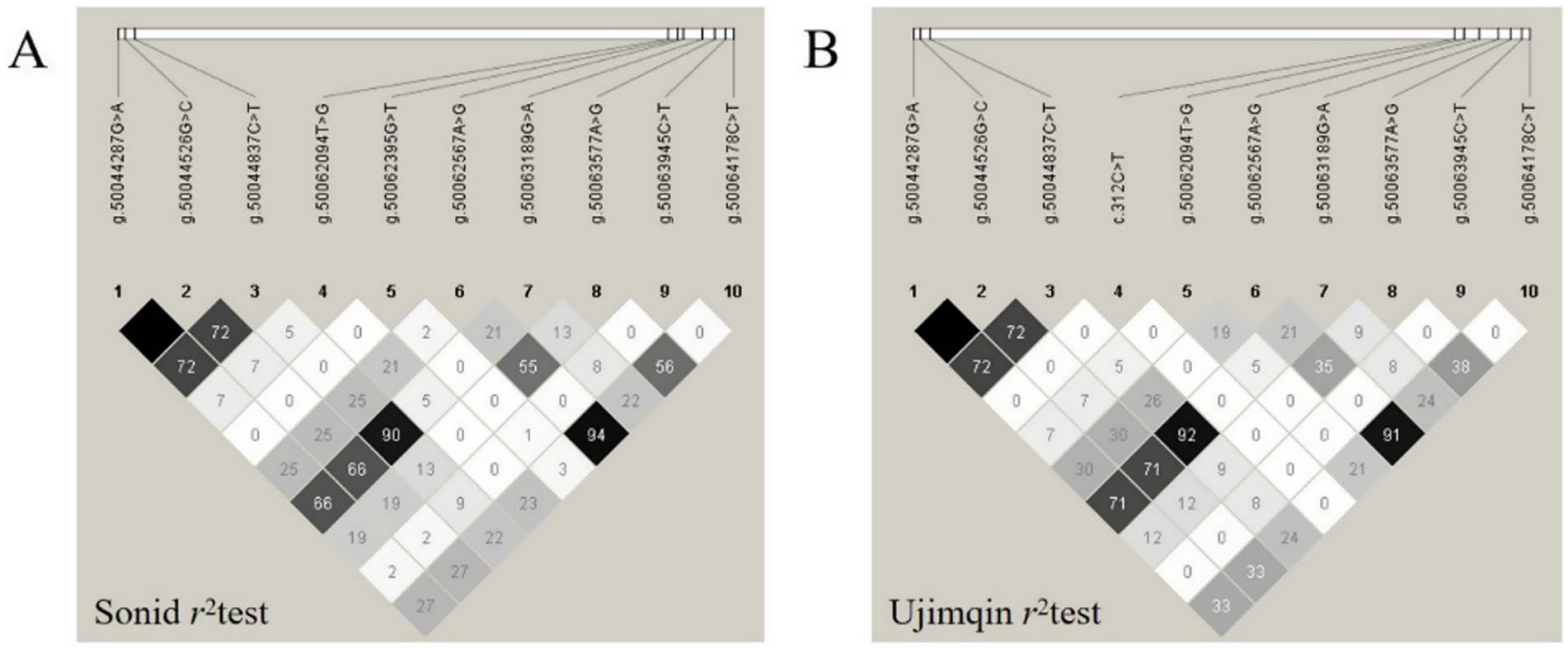
Linkage disequilibrium of *TGF-β1* variants in Sonid and Ujimqin sheep. **(A)** Sonid sheep. **(B)** Ujimqin sheep. *r^2^* values indicate pairwise LD strength.

LD (estimated as the *r*^2^) was calculated for the 11 SNPs in both Sonid and Ujimqin sheep. In this study, we define complete LD with *r*^2^ = 1.00, and strong LD with *r*^2^ > 0.70. The obtained *r^2^* values indicated complete LD between g.50044287 G > A and g.50044526 G > C in the population of 379 individuals across both experimental sheep breeds. Therefore, these LD blocks were analyzed together and designated as Sonid-LD and Ujimqin-LD. Meanwhile, g.50044837 C > T showed strong LD with each of the Sonid-LD and Ujimqin-LD blocks (Figure 3; Tables 3, 4).

**Table 3:** Linkage disequilibrium (D*′* and *r^2^*) among variants in Sonid sheep population.

D′/*r*^2^	g.50062094T > G	g.50062395G > T	g.50062567 A > G	g.50063189G > A	g.50063577 A > G	g.50063945 C > T	g.50064178 C > T	g.50044287G > A	g.50044526G > C	g.50044837 C > T
g.50062094 T > G		0.000	0.213	0.052	0.000	0.002	0.238	0.073	0.073	0.053
g.50062395 G > T	1.000		0.028	0.007	0.000	0.015	0.031	0.002	0.002	0.007
g.50062567 A > G	1.000	1.000		0.210	0.551	0.009	0.943	0.255	0.255	0.25
g.50063189 G > A	1.000	1.000	0.931		0.134	0.088	0.223	0.669	0.669	0.902
g.50063577 A > G	0.383	1.000	1.000	1.000		0.005	0.564	0.190	0.190	0.138
g.50063945 C > T	1.000	0.441	1.000	1.000	1.000		0.008	0.026	0.026	0.09
g.50064178 C > T	1.000	1.000	1.000	1.000	1.000	1.000		0.278	0.278	0.227
g.50044287 G > A	1.000	1.000	0.859	0.975	1.000	1.000	0.938		1.000	0.723
g.50044526 G > C	1.000	1.000	0.859	0.975	1.000	1.000	0.938	1.000		0.723
g.50044837 C > T	1.000	1.000	1.000	0.963	1.000	1.000	1.000	1.000	1.000	

**Table 4:** Linkage disequilibrium (D*′* and *r^2^*) among variants in Ujimqin sheep population.

D′/*r*^2^	c.312 C > T	g.50062094 T > G	g.50062567 A > G	g.50063189 G > A	g.50063577 A > G	g.50063945 C > T	g.50064178 C > T	g.50044287G > A	g.50044526G > C	g.50044837 C > T
c.312 C > T		0.000	0.001	0.002	0.000	0.000	0.001	0.002	0.002	0.002
g.50062094 T > G	1.000		0.195	0.052	0.002	0.002	0.213	0.070	0.070	0.051
g.50062567 A > G	1.000	1.000		0.214	0.355	0.005	0.916	0.301	0.301	0.263
g.50063189 G > A	1.000	1.000	0.895		0.095	0.088	0.244	0.710	0.710	0.928
g.50063577 A > G	1.000	1.000	1.000	1.000		0.002	0.388	0.128	0.128	0.094
g.50063945 C > T	1.000	1.000	0.738	1.000	0.878		0.003	0.005	0.005	0.087
g.50064178 C > T	1.000	1.000	1.000	1.000	1.000	0.577		0.331	0.331	0.241
g.50044287 G > A	1.000	1.000	0.913	0.981	1.000	0.459	1.000		1.000	0.728
g.50044526 G > C	1.000	1.000	0.913	0.981	1.000	0.459	1.000	1.000		0.728
g.50044837 C > T	1.000	1.000	1.000	0.970	1.000	1.000	1.000	1.000	1.000	

### 3.4 Association between novel *TGF-β1* gene variants and litter size

The associations of 11 variants in the *TGF-β1* gene with litter size were evaluated in 231 Sonid ewes (Table 5) and 148 Ujimqin ewes (Table 6). Significant associations exist between TGF-β1 3′UTR variants g.50044287 G > A, g.50044526 G > C, g.50044837 C > T and litter size in Sonid ewes (*p* < 0.01; Table 5). Furthermore, g.50044287 G > A and g.50044526 G > C are significantly associated with litter size in Ujimqin ewes (*p* < 0.05; Table 6).

**Table 5:** The effects of the genotypes of the 11 *TGF-β1* variants on litter size in the Sonid sheep population.

Variant	Genotype	Number	Litter size
g.50062094 T > G	TG	13	1.23 ± 0.12
	GG	217	1.15 ± 0.03
g.50062567 A > G	AG	51	1.22 ± 0.06
	GG	174	1.13 ± 0.03
g.50063189 G > A	GG	43	1.21 ± 0.07
	GA	102	1.15 ± 0.06
	AA	85	1.16 ± 0.04
g.50063577 A > G	AG	35	1.22 ± 0.07
	GG	195	1.14 ± 0.03
g.50063945 C > T	CC	206	1.17 ± 0.03
	CT	25	1.08 ± 0.06
g.50064178 C > T	CT	45	1.24 ± 0.06
	TT	177	1.13 ± 0.02
g.50044287 G > A of Sonid-LD	GG	22	1.54 ± 0.11A
	GA	101	1.15 ± 0.04B
	AA	108	1.08 ± 0.03B
g.50044837 C > T	CC	32	1.36 ± 0.09A
	CT	115	1.14 ± 0.04B
	TT	84	1.08 ± 0.03B

Different capital letters in the same group mean significant difference A, B: *p* < 0.01.

**Table 6:** The effects of the genotypes of the 11 *TGF-β1* variants on litter size in the Ujimqin sheep population.

Variant	Genotype	Number	Litter size
g.50062567 A > G	AG	37	1.20 ± 0.42
	GG	109	1.20 ± 0.40
g.50063189 G > A	GG	14	1.10 ± 0.18
	GA	79	1.24 ± 0.18
	AA	55	1.16 ± 0.05
g.50063577 A > G	AG	16	1.13 ± 0.09
	GG	132	1.20 ± 0.04
g.50063945 C > T	CC	133	1.21 ± 0.04
	TT	15	1.07 ± 0.07
g.50064178 C > T	CT	34	1.18 ± 0.07
	TT	112	1.21 ± 0.04
g.50044287 G > A of Ujimqin-LD	GG	14	1.43 ± 0.14a
	GA	63	1.19 ± 0.05b
	AA	71	1.15 ± 0.04b
g.50044837 C > T	CC	21	1.33 ± 0.11
	CT	70	1.19 ± 0.05
	TT	57	1.16 ± 0.05

Different small letters in the same group mean significant difference a, b: *p* < 0.05.

### 3.5 Bioinformatics analysis of ovine *TGF-β1* gene

#### 3.5.1 Characteristics and structural prediction of ovine TGF-*β*1 protein

**Figure 4:**
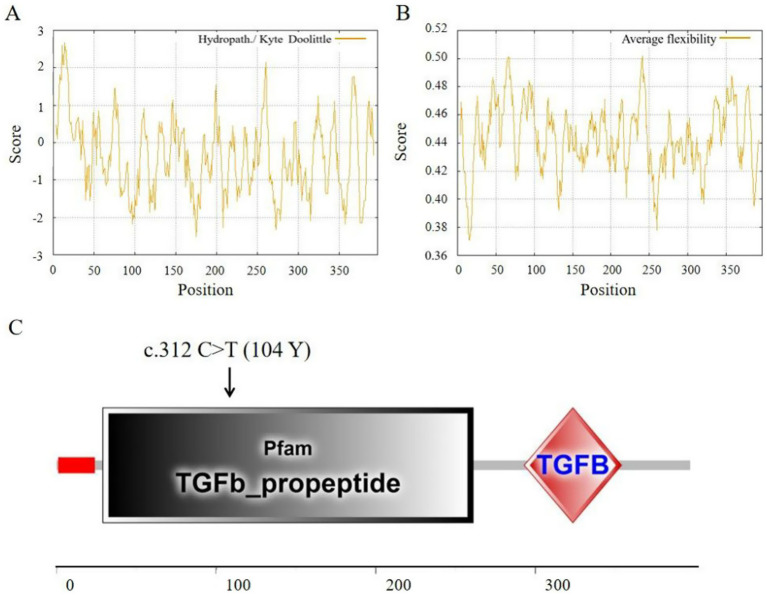
Structural features of ovine TGF-β1 protein. **(A)** Hydropathy profile. **(B)** Flexibility index. **(C)** Predicted conserved structural domains.

Hydropathy analysis of the sheep TGF-β1 protein revealed a maximum hydrophobicity value of 0.950 at position 14 aa (amino acids) and a minimum value of −0.872 at position 97 aa (Figure 4A). The maximum average flexibility index was observed at position 241 aa (0.502), while the minimum average flexibility index occurred at positions 16 aa and 17 aa (0.371) (Figure 4B). The SMART (Simple Modular Architecture Research Tool) was employed to predict conserved structural domains. A signal peptide was identified in the range of 1–24 amino acids (aa). The TGF-β propeptide region, which forms the LAP (Latent Associated Peptide) motif within the TGFb_propeptide domain, was localized to positions 29–261 aa. Additionally, the TGFB (TGF-β) domain was mapped to residues 293–354 aa (Figure 4C).

#### 3.5.2 Amino acid sequence analysis of ovine TGF-β1

**Figure 5:**
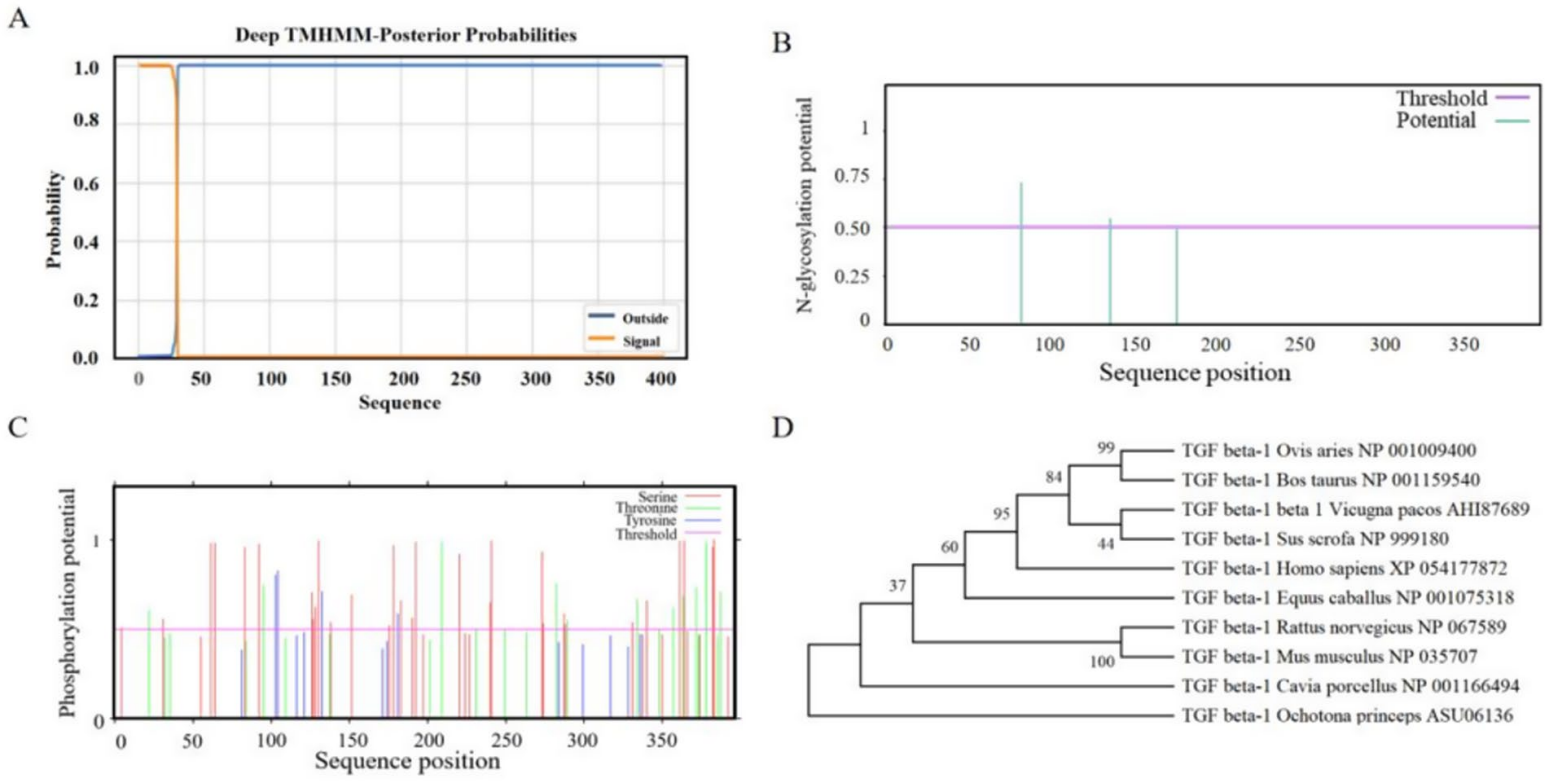
Predicted structural and functional features of ovine TGF-β1 protein. **(A)** Transmembrane helices. **(B)** N-glycosylation sites. **(C)** Phosphorylation sites. **(D)** Phylogenetic tree of TGF-β1 homologous sequences.

Physicochemical properties of the ovine TGF-β1 protein sequence were predicted using ProtParam. The molecular formula of *TGF-β1* is C₁₉₉₁H₃₁₆₇N₅₆₇O₅₈₄S₁₈, with a molecular weight of 44,968.58 Da and an isoelectric point of 8.91. Amino acid composition analysis revealed that leucine exhibits the highest proportion (13.6%) while tryptophan shows the lowest (1.8%). The total number of negatively charged amino acid residues (Asp + Glu) was 43, exceeding the count of positively charged residues (Arg + Lys) at 51. DeepTMHMM was employed to predict transmembrane domains, identifying positions 1–29 aa as the transmembrane region (Figure 5A). NetOGlyc and NetPhos were utilized for post-translational modification predictions: three N-glycosylation sites were detected at Asn 82, Asn 136, and Asn 176, while 44 phosphorylation sites were predicted, comprising 30 Ser, 10 Thr, and 4 Tyr residues (Figures 5B, C).

#### 3.5.3 Multi-sequence alignment and phylogenetic analysis

To construct a phylogenetic tree for sheep *TGF-β1* homologs, amino acid sequences from nine species were retrieved from NCBI, including sheep (*Ovis aries*), cattle (*Bos taurus*), alpaca (*Vicugna pacos*), *Homo sapiens*, guinea pig (*Cavia porcellus*), domestic horse (*Equus caballus*), *Rattus norvegicus*, *Mus musculus*, wild boar (*Sus scrofa*), and Pikas (Ochotona) hare. A neighbor-joining phylogenetic tree was generated using MEGA software (Version X) with 1,000 bootstrap replicates. The analysis demonstrated high sequence homology between sheep and cattle *TGF-β1* proteins, whereas lower homology was observed between sheep and North American snowshoe hare (Figure 5D).

#### 3.5.4 Impact of ovine *TGF-β1* gene variants on mRNA secondary structure

**Figure 6:**
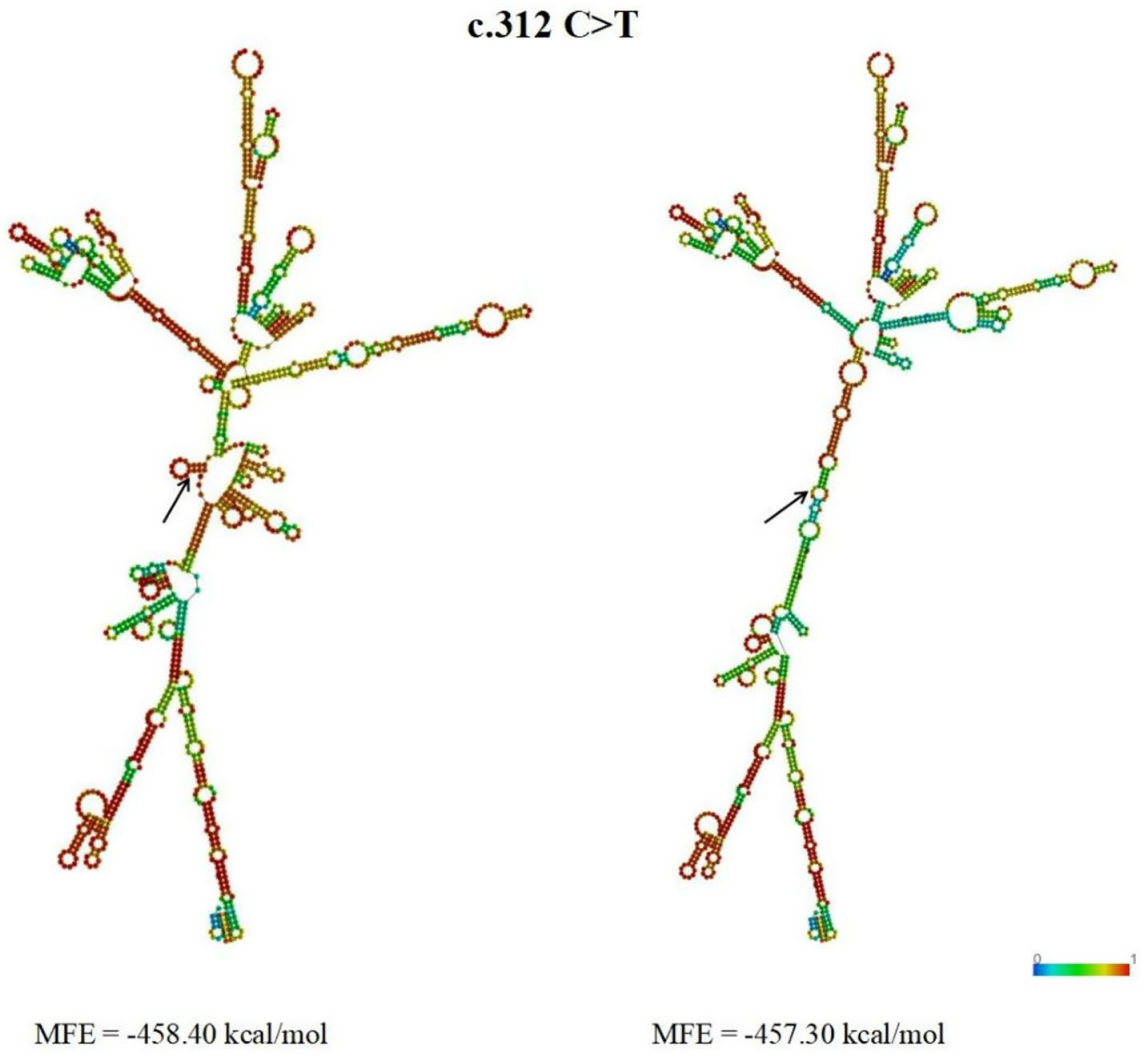
mRNA secondary structure and MFE for wild-type and mutant *TGF-β1*. Structures depict base-pairing probabilities.

The secondary structure and minimum free energy (MFE) of TGF-β1 mRNA were predicted using RNAfold. The c.312 C > T mutation in exon 1 altered its secondary structure significantly, with a concomitant MFE increase from −458.40 to −457.30 kcal/mol (ΔMFE = +1.10 kcal/mol) (Figure 6).

#### 3.5.5 Prediction of miRNA binding sites in the 3′UTR of ovine *TGF-β1* gene

**Figure 7:**
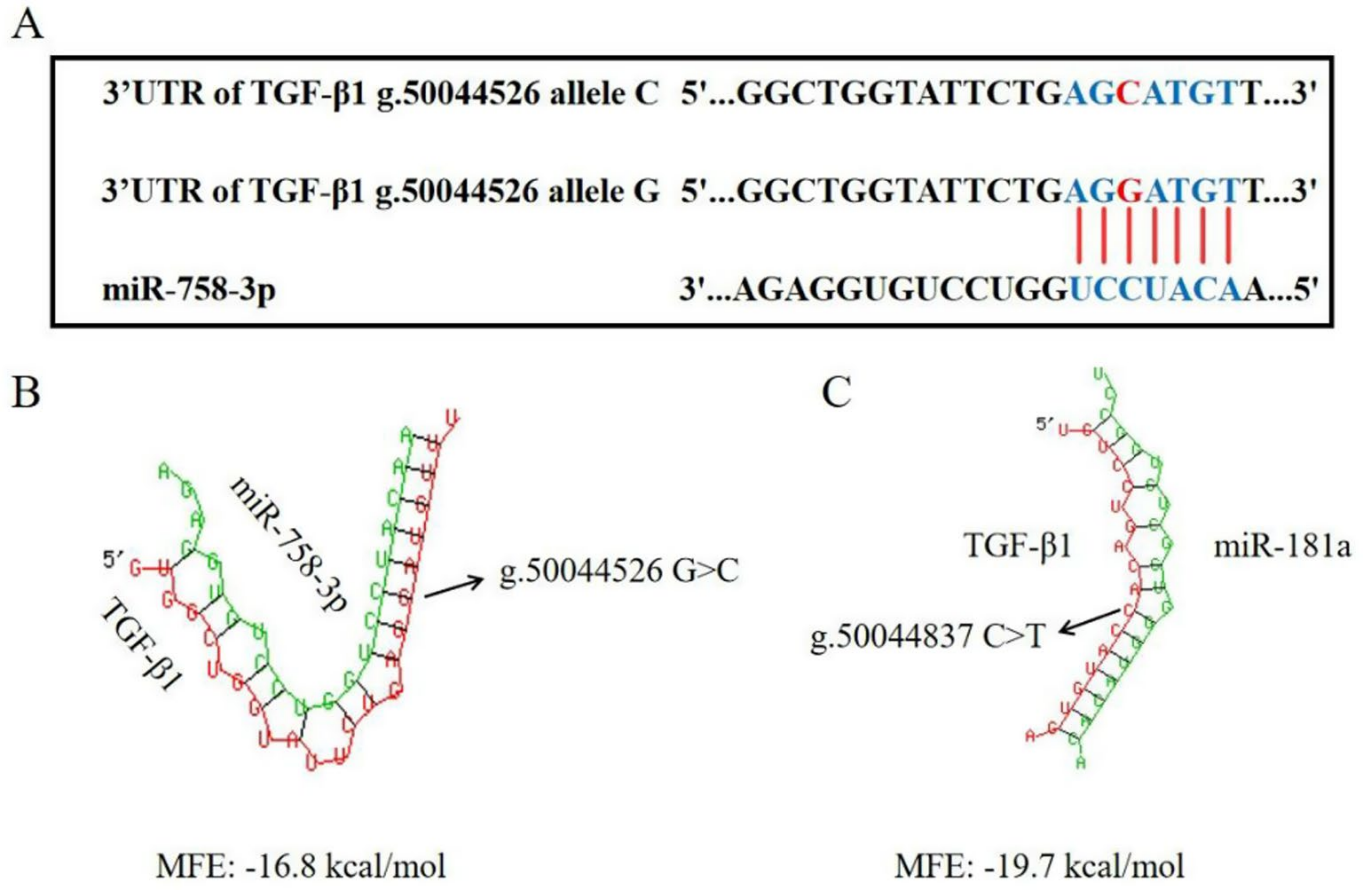
Predicted binding sites for miRNAs in the *TGF-β1* 3′UTR. **(A)** miRanda: miR-758-3p at G allele. **(B)** RNAhybrid: miR-758-3p at G allele. **(C)** RNAhybrid: miR-181 at C allele.

We utilized dual bioinformatic algorithms (miRanda and RNAhybrid) to predict miRNAs targeting the functional regulatory variants g.50044287 G > A, g.50044526 G > C, and g.50044837 C > T within the TGF-β1 3′UTR (Figures 7A–C). Bioinformatic analysis revealed perfect seed-sequence complementarity between miR-758-3p and both the canonical TGF-β1 3′UTR and the G allele of identified regulatory variants (Figures 7A,B), demonstrating its function as a key *cis*-regulatory element that mediates allele-specific suppression of TGF-β1 expression through direct binding to G allele-containing transcripts.

## 4 Discussion

As a crucial cytokine, TGF-β1 plays significant roles in various reproductive regulations, including embryonic development (36), follicular development and ovulation (37), granulosa cell luteinization (38), tissue fibrosis (39), and immune responses (40). Studies on the ovaries of Small-tailed Han sheep revealed that *TGF-β1* regulates ovarian granulosa cell proliferation and participates in follicular development through the classical downstream signaling pathway involving SMAD factors (19). Additionally, the TGF-β1 signaling pathway modulates ovarian granulosa cell apoptosis and follicle atresia (36, 41). These findings highlight the relationship between the *TGF-β1* gene and ovarian function. However, limited research has been conducted on Ujimqin sheep and Sonid sheep regarding TGF-β1. Eleven novel *TGF-β1* variants were identified in Sonid and Ujimqin sheep, facilitating investigations into associations with litter size and elucidation of the gene’s function.

The 3′untranslated region serves as a critical regulatory hub within mRNA non-coding sequences, governing post-transcriptional gene expression through various control elements (42). Polymorphism (g.42314637 T > C) in the 3′UTR of the *SF1* gene can increase litter size by modulating gene stability in Small-tailed Han sheep (43). In Hu sheep, direct miRNA-TGF-*β* pathway interactions have been documented to modulate granulosa cell dynamics and follicular development (44). Notably, the 3′UTR of *BAMBI* mRNA exhibits functional miR-19a-3p binding capacity that potentiates TGF-β1/SMAD2/3 signaling (45). Clinical studies further reveal that miR-758-3p-mediated suppression of *DCUN1D1* via 3′UTR binding promotes cervical cancer progression through analogous mechanisms (46). Our investigation identified three novel *TGF-β1* 3′UTR variants (g.50044287 G > A, g.50044526 G > C, and g.50044837 C > T) significantly associated with litter size in Sonid sheep. Intriguingly, two variants (g.50044287 G > A and g.50044526 G > C) demonstrated conserved association in Ujimqin sheep populations. We therefore propose that the polymorphic site (g.50044526 G > C) in the 3′UTR of the ovine *TGF-β1* gene serves as a functional binding site for miR-758-3p. This 3′UTR mutation likely reduces both mRNA stability and protein expression levels of *TGF-β1*, thereby suppressing TGF-β signaling activity in ovarian cells and ultimately contributing to decreased litter size in sheep. Furthermore, the complete LD observed among the three mutations (g.50044287 G > A, g.50044526 G > C, and g.50044837 C > T) implies that the g.50044287 G > A and g.50044837 C > T variants may synergistically influence the functional impact of g.50044526 G > C through coordinated regulatory mechanisms, collectively affecting reproductive outcomes. However, this hypothesis requires direct experimental validation through a dual-luciferase reporter assay comparing the constructs with the G and C alleles in sheep granulosa cells. Future studies should verify the association between mutations and litter size in large sheep population.

The minimum free energy of the silenced c.312 C > T mutation increased from −458.40 (wild-type) to −457.30 (mutation) kcal/mol. This MFE elevation implies reduced stability of the mRNA secondary structure (47). Studies confirm that single base-pair substitutions can modify mRNA secondary structure and stability (48, 49). Although synonymous mutations preserve amino acid sequences, they impact multiple gene expression processes—including transcription, mRNA processing, translation, and co-translational folding (50). Notably, the c.312 C > T synonymous mutation was observed in only one Ujimqin sheep and thus excluded from the litter size association analysis. Hence, any conclusion about its effect on mRNA structure is presently hypothetical and awaits confirmation in larger cohorts.

The Ujimqin sheep is a typical Mongolia sheep breed. In our previous studies, it was found that variants including *BMPR1B* (NC_056059.1) c.687 G > A (51), *LEPR* (NC_040252.1) variants c.240 C > T/c.279 C > T (35), and *BMP15* (NC_019484.2) promoter variants g.50988478 C > A and g.50987863 G > A (31) collectively regulate the litter size of Mongolia sheep. Furthermore, ongoing investigations reveal associations between *TGF-β1* variants and litter size in two Mongolia sheep breeds: Ujimqin (g.50044287 G > A and g.50044287 G > C) and Sonid (g.50044287 G > A, g.50044526 G > C, g.50044837 C > T). These populations exhibit considerable genetic diversity in reproductive traits within the Mongolia sheep lineage (35, 52). We therefore hypothesize that litter size in these breeds may be regulated by combinatorial effects of multiple genes, analogous to the genetic architecture observed in Romanov sheep (53). Integration of these results positions *TGF-β1* as a prime candidate for MAS in low-fecundity breeds including Sonid and Ujimqin sheep.

## 5 Conclusion

Our study demonstrates that in Sonid and Ujimqin ewes, the g.50044287 G > A and g.50044526 G > C variants in 3′UTR of the *TGF-β1* gene are in complete LD and show significant associations with litter size, respectively. The 3′UTR variant g.50044837 C > T exhibits strong LD with both the Sonid-LD1 and Ujimqin-LD1 blocks and is additionally associated with litter size in Sonid sheep. These markers could be potentially utilized in MAS to enhance litter size in the Sonid sheep and Ujimqin sheep. These results not only provide valuable genetic markers for sheep breeding programs through their demonstrated association with reproductive performance, but also contribute novel mutation resources for functional studies of the *TGF-β1* gene. However, before considering their application in commercial breeding systems, it is imperative that the effect sizes and predictive accuracy of these markers be further validated in larger, independent populations.

## Data Availability

The novel insights offered by this research are documented within the article and supplementary materials; and the variant data for this study also have been deposited in the European Variation Archive (EVA) at EMBL-EBI under accession number PRJEB106333, https://www.ebi.ac.uk/eva/?eva-study=PRJEB106333.

## Footnotes

1
http://www.agenabio.com/assay-design-suite-20-software

2
https://web.expasy.org/protparam/

3
https://doi.org/10.1101/2022.04.08.487609

4
https://services.healthtech.dtu.dk/services/NetPhos-3.1/

5
https://web.expasy.org/protscale/

6
https://services.healthtech.dtu.dk/services/NetOGlyc-4.0/

7
http://rna.tbi.univie.ac.at//cgi-bin/RNAWebSuite/RNAfold.cgi

8
https://npsa.lyon.inserm.fr/cgi-bin/npsa_automat.pl?page=/NPSA/npsa_sopma.html

9
https://www.uniprot.org/align

10
https://www.mirbase.org/
