# Supplementary material for: Polymorphisms in the 3′UTR of the TGF-β1 gene associated with litter size in Ujimqin and Sonid sheep
Source: Front Vet Sci. 2026 Feb 11;12:1700201. doi: 10.3389/fvets.2025.1700201 (PMC12933948; doi:10.3389/fvets.2025.1700201)
Supplement: Supplementary file 1 [file Table_1.doc]

| **Supplementary Table S1** | | | |
| --- | --- | --- | --- |
| Supplementary Table 1. MassARRAY primers used for genotyping of eleven variants in *TGF-β1* gene. | | | |
| **Name** | **Target Region** | **Primer Sequence (5'-3')** | **Annealing Temperature (°C)** |
| c.312 | CDS 1 | F: ACGTTGGATGTTTACAACAGTACCCGCGAC | 46.5 |
|  |  | R: ACGTTGGATGGTATTCCACCATTAGCACGC |  |
|  |  | E: CAGAGGCGGACTACTA |  |
| g.50063189 | Promoter | F: ACGTTGGATGTCTTTATGCTCTCGCTGACC | 56.1 |
|  |  | R: ACGTTGGATGACCAACAGGGTGCTGCCTC |  |
|  |  | E: CACCCACAAGCATCACTGCC |  |
| g.50063577 | Promoter | F: ACGTTGGATGAATGGGCTAGTAGGTGTGTG | 48 |
|  |  | R: ACGTTGGATGGGTCAAAAAAGCACCCACAC |  |
|  |  | E: GAAGGGCTCAATAAAGATGT |  |
| g.50063945 | Promoter | F: ACGTTGGATGCATGGAGATGCCATCTACAG | 49.9 |
|  |  | R: ACGTTGGATGACCCCATACCGATCAAAGTG |  |
|  |  | E: GAAATCTACAGTGGGGCTGA |  |
| g.50064178 | Promoter | F: ACGTTGGATGACTGTACCCCTTCACCTTTC | 48.4 |
|  |  | R: ACGTTGGATGCAAAAGCCATAGCCTGCAAG |  |
|  |  | E: CCTTTCCACCCTCCC |  |
| g.50062094 | 5'flanking region | F: ACGTTGGATGTCTTCCCACCCCAGTCCAG | 66.5 |
|  |  | R: ACGTTGGATGCCAGGCGTCAGCATTAGTAG |  |
|  |  | E: AAGCTCGCGCTCTCGGCTGTGCC |  |
| g.50062395 | 5'flanking region | F: ACGTTGGATGAAGACTTGACCCCAGACTTC | 65.4 |
|  |  | R: ACGTTGGATGATCCCGGATATCGGAGAAGG |  |
|  |  | E: AGCCTCTCTCCTGAGCCCCCGC |  |
| g.50062567 | 5'flanking region | F: ACGTTGGATGTTTGCGGTTCCTGATGGCTG | 51.9 |
|  |  | R: ACGTTGGATGAACTTGAGGACCCCAGACAG |  |
|  |  | E: AAGCAAGCGTCCGAGG |  |
| g.50044287 | 3'untranslated region | F: ACGTTGGATGGAGACACCAGAGCACAAATG | 52.2 |
|  |  | R: ACGTTGGATGTGCTTTGGAGTTTTCGGTGG |  |
|  |  | E: ATGCAGCCCTGCACA |  |
| g.50044526 | 3'untranslated region | F: ACGTTGGATGGAGACACCAGAGCACAAATG | 46.9 |
|  |  | R: ACGTTGGATGTTCGTGATCAGTGTGTGTGG |  |
|  |  | E: CCCCACAGATACAAACAT |  |
| g.50044837 | 3'untranslated region | F: ACGTTGGATGAAATAACACACCCCAGGGAC | 52.1 |
|  |  | R: ACGTTGGATGTTCTGCTGTACAAGCTGGTG |  |
|  |  | E: AAAAGGCTAACCGAGTCACATG |  |
| Note: CDS: coding region, F: forward primer sequence, R: reverse primer sequence, E: extended primer sequence. | | | |
