# Supplementary material for: Polymorphisms in the 3′UTR of the TGF-β1 gene associated with litter size in Ujimqin and Sonid sheep
Source: Front Vet Sci. 2026 Feb 11;12:1700201. doi: 10.3389/fvets.2025.1700201 (PMC12933948; doi:10.3389/fvets.2025.1700201)
Supplement: Supplementary file 2 [file Table_2.doc]

| Supplementary Table 2.Genotype groups with fewer than ten of eleven variants in *TGF-β1* gene. | | | |
| --- | --- | --- | --- |
| Sheep breeds | Variant | Genotype | Number |
| Sonid | g.50062094 T>G | TT | 1 |
|  | g.50062567 A>G | AA | 6 |
|  | g.50063577 A>G | AA | 1 |
|  | g.50062395 A>G | GT | 2 |
|  | g.50064178 C>T | CC | 6 |
| Ujimqin | c.312 | CT | 1 |
|  | g.50062567 A>G | AA | 2 |
|  | g.50064178 C>T | CC | 2 |
